# Supplementary material for: Der f 38 Is a Novel TLR4-Binding Allergen Related to Allergy Pathogenesis from Dermatophagoides farinae
Source: Int J Mol Sci. 2021 Aug 5;22(16):8440. doi: 10.3390/ijms22168440 (PMC8395149; doi:10.3390/ijms22168440)
Supplement: Supplementary file 1 [file ijms-22-08440-s001.zip › ijms-1310593-supplementary.pdf]

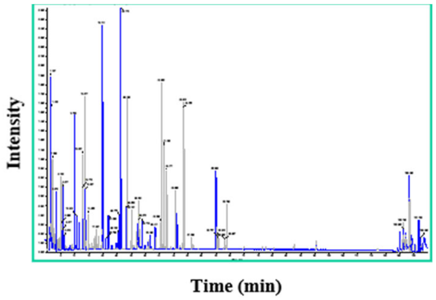

Figure S1. Identification of native Der f 38. Mass spectrum of natural Der f 38 protein after chymotrypsin treatment. The Q-TOF method (ABsciex TripleTOF 5600+) was used for the analysis of Der f 38. X-axis shows the mass/charge ratio and Y-axis represents signal intensity.

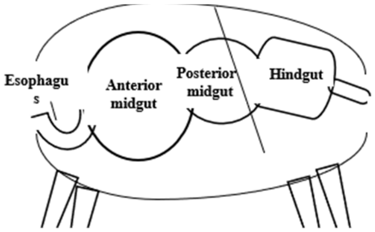

Figure S2. Illustration of the digestive system of DF. Line indicates the region cut from the digestive tract in electron microscopy.

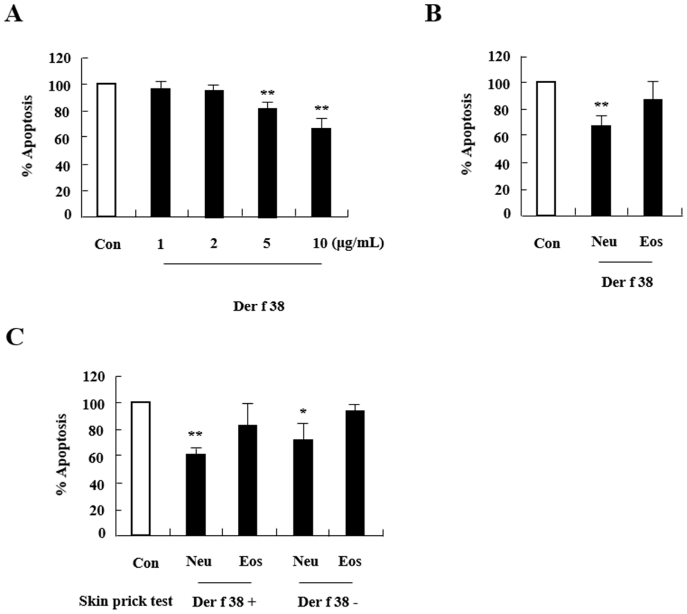

Figure S3. Der f 38 inhibits apoptosis of neutrophils and eosinophils. (A) Normal neutrophils were incubated with Der f 38 at the indicated concentration for 24 h. (B) After treatment with Der f 38 (10 μg/mL), % apoptotic rate of neutrophils and eosinophils of normal (n = 3) was combined with that of allergic subjects (n = 8), respectively. (C) Date from allergic subjects (n = 8) were divided into Der f 38-positive (n = 6) or -negative (n = 2) results based on skin prick test. *, $p < 0.05$; **, $p < 0.01$, significant difference between untreated and Der f 38-treated groups.

Supplementary Table S1. Characteristics of normal and allergic subjects

	Normal	Allergy
Number of subjects (female/male)	20 (15/5)	20 (15/5)
Age (years)	23.9 ± 5.0 (20~41)	23.9 ± 5.8 (20~48)
RBC (10^6/μL)	4.6 ± 0.3 (4.0~5.4)	4.8 ± 0.4 (4.3~5.8)
Platelet (10^3/μL)	294.3 ± 52.7 (220~384)	301.0 ± 79.6 (149~483)
WBC (10^3/μL)	6.4 ± 1.4 (4.5~9.3)	6.8 ± 1.1 (4.4~8.5)
Neutrophil (%)	56.2 ± 8.0 (36.4~72.1)	56.9 ± 8.5 (35.8~66.1)
Lymphocyte (%)	36.2 ± 7.8 (27.2~58.4)	33.0 ± 6.9 (22.8~47.7)
Monocyte (%)	6.4 ± 1.4 (3.7~9.4)	7.4 ± 1.9 (5.1~11.8)
Eosinophil (%)	1.2 ± 0.8 (0.2~2.8)	2.0 ± 1.0 (0.6~3.7)
Basophil (%)	0.4 ± 0.2 (0.1~0.9)	0.5 ± 0.2 (0.1~0.9)
Total IgE (ng/mL)	81.8 ± 22.7 (55.3~121.4)	691.6 ± 552.8 (42.4~1594.5)
